# ETS1 Mediates MEK1/2-Dependent Overexpression of Cancerous Inhibitor of Protein Phosphatase 2A (CIP2A) in Human Cancer Cells

**DOI:** 10.1371/journal.pone.0017979

**Published:** 2011-03-22

**Authors:** Anchit Khanna, Juha Okkeri, Turker Bilgen, Timo Tiirikka, Mauno Vihinen, Tapio Visakorpi, Jukka Westermarck

**Affiliations:** 1 Institute of Medical Technology, University of Tampere and Tampere University Hospital, Tampere, Finland; 2 Tampere Graduate Program in Biomedicine and Biotechnology (TGPBB), University of Tampere, Tampere, Finland; 3 Turku Centre for Biotechnology, University of Turku and Åbo Akademi University, Turku, Finland; 4 Department of Medical Biology and Genetics, Faculty of Medicine, Akdeniz University, Antalya, Turkey; 5 Department of Pathology, University of Turku, Turku, Finland; SanfordBurnham Medical Research Institute, United States of America

## Abstract

EGFR-MEK-ERK signaling pathway has an established role in promoting malignant growth and disease progression in human cancers. Therefore identification of transcriptional targets mediating the oncogenic effects of the EGFR-MEK-ERK pathway would be highly relevant. Cancerous inhibitor of protein phosphatase 2A (CIP2A) is a recently characterized human oncoprotein. CIP2A promotes malignant cell growth and is over expressed at high frequency (40–80%) in most of the human cancer types. However, the mechanisms inducing its expression in cancer still remain largely unexplored. Here we present systematic analysis of contribution of potential gene regulatory mechanisms for high CIP2A expression in cancer. Our data shows that evolutionary conserved CpG islands at the proximal CIP2A promoter are not methylated both in normal and cancer cells. Furthermore, sequencing of the active CIP2A promoter region from altogether seven normal and malignant cell types did not reveal any sequence alterations that would increase CIP2A expression specifically in cancer cells. However, treatment of cancer cells with various signaling pathway inhibitors revealed that CIP2A mRNA expression was sensitive to inhibition of EGFR activity as well as inhibition or activation of MEK-ERK pathway. Moreover, MEK1/2-specific siRNAs decreased CIP2A protein expression. Series of CIP2A promoter-luciferase constructs were created to identify proximal −27 to −107 promoter region responsible for MEK-dependent stimulation of CIP2A expression. Additional mutagenesis and chromatin immunoprecipitation experiments revealed ETS1 as the transcription factor mediating stimulation of CIP2A expression through EGFR-MEK pathway. Thus, ETS1 is probably mediating high CIP2A expression in human cancers with increased EGFR-MEK1/2-ERK pathway activity. These results also suggest that in addition to its established role in invasion and angiogenesis, ETS1 may support malignant cellular growth via regulation of CIP2A expression and protein phosphatase 2A inhibition.

## Introduction

Accumulation of various genetic alterations has been considered as a prerequisite for cancer development. These genetic alterations often results in overexpression or activity of proto-oncogenes and inhibition of the function of tumor suppressor [1], [2].Therefore, understanding of the mechanisms by which the activity of both proto-oncogenes and tumor suppressors is altered in cancer is crucially important both academically, and for development of new approaches to target cancer cells for therapy.

Epidermal growth factor receptor (EGFR)-mediated MEK1/2-ERK MAPK pathway activity has been shown to regulate virtually all aspects involved in tumourigenesis. Accordingly, increased activity and overexpression of both EGFR and the MEK1/2 kinases has been observed in various human cancers [3],[4],[5],[6]. Moreover, inhibitors for EGFR, Raf and MEK1/2 kinases are in clinical trials against various types of solid tumors [3], [4], [7], [8]. Interestingly, increased MEK1/2 pathway activity due to hyperactivity of Ras and Raf proteins has also shown to contribute to clinical resistance to EGFR tyrosine kinase inhibitor [4], [9], [10]. These results together suggest that inhibition of the pathway activity both at the level of the receptor, and its downstream effectors may be required for an effective anti-cancer therapy.

ETS family of transcription factors including Elk1, ETS1 and ETS2 are some of the well-known targets for the EGFR-Ras-MEK1/2 signaling pathway [11]. ETS1 and ETS2 are both phosphorylated by Ras signaling [11], [12]. ETS1 is a founding family member of ETS-domain transcription factors. It has been linked to cancer since its identification as an oncogenic fusion with the product of c-Myb proto-oncogene in the E26 avian leukemia virus [13], [14]. ETS1 is known to target a wide variety of genes [11], [12], [15], which in turn dictates its role in various cellular processes. Pertaining to cancer ETS1 is best known for its role in promoting tumor cell invasiveness, motility and metastasis [13], [16]. Invasion promoting role of ETS1 is thought to be mediated by transcriptional up regulation of genes that participate on degradation of extracellular matrix and stimulation of angiogenesis [16]. Interestingly, even though ETS1 and other ETS-family transcription factors have been mainly linked to tumor invasion, soon after cloning of human ETS1, Seth and collaborators demonstrated that ETS1 overexpression transformed NIH3T3 cells making them capable of anchorage-independent growth and tumor growth in nude mice [17]. More recently it was also shown that ETS1 promoted transformed cellular phenotype in human cells as well [18], [19]. However, the target genes involved in the ETS1-mediated cellular transformation are poorly understood.

Cancerous Inhibitor of Protein Phosphatase 2A (CIP2A) is a recently characterized human oncoprotein [20]. CIP2A interacts with and inhibits protein phosphatase 2A (PP2A) tumor suppressor complex and thereby inhibits dephosphorylation and subsequent proteolytic degradation of MYC transcription factor [20], [21]. CIP2A promotes Ras-elicited foci formation in mouse embryo fibroblasts and supports transformation of immortalized human cells [20]. In loss of function studies, CIP2A depletion has been shown to reduce the overall tumor xenograft size in nude mice [20], [22], and to impair clonogenicity and anchorage-independent growth of tumor cells [20], [22], [23], [24], [25]. Recently, CIP2A was also shown to inhibit Akt kinase-associated PP2A activity and by these means to protect human hepatocellular carcinoma cells from bortezomib-induced apoptosis [26]. CIP2A is expressed in only very few normal tissues but it is overexpressed with very high incidence (40–80%) in various human cancer types such as head and neck squamous cell carcinomas (HNSCC), colon carcinomas, gastric carcinomas, breast carcinomas and non-small cell lung cancer [20], [22], [23],[24],[25]. In addition to its overexpression in cancers, recent studies have shown that CIP2A immunopositivity correlates with aggressive disease and/or poor patient survival in several cancer types [22], [23], [24]. With respect to mechanisms regulating CIP2A expression, we have hitherto identified MYC as a stimulator of CIP2A expression [24]. However, other mechanisms contributing towards increased CIP2A expression in human cancers still remain elusive.

In the current study, we cloned functional CIP2A promoter region and identified the promoter regions mediating high CIP2A transcriptional activity. In addition, using chemical inhibitors for various signaling pathways and target specific siRNAs, we dissected the role of EGFR-MEK1/2 pathway in regulating CIP2A expression. Chromatin immunoprecipitation, promoter mutagenesis and target specific siRNAs were then utilized to identify ETS1 as the transcription factor regulating EGFR-MEK1/2-dependent CIP2A expression in human cancers.

## Results

### Bioinformatic analysis and methylation status of CIP2A Promoter

**Figure 1:**
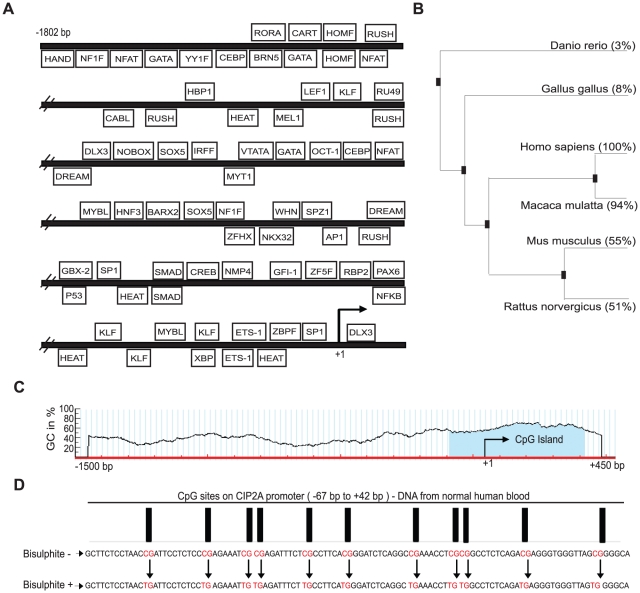
Bioinformatic analysis and methylation status of CIP2A Promoter. A. Identification of transcription factor binding sites with matrix similarity of 95% and core similarity of 100% on −1802 bp CIP2A promoter using Genomatix software. B. Phylogenetic tree depicting the evolutionary conservation of CIP2A promoter. C. Identification of putative CpG Island from −150 bp to +400 bp (blue shaded area) on the CIP2A promoter using MethPrimer software. D. Shows the sequencing results of the extracted genomic DNA from normal human blood. All CpG sites (represented by black rectangular blocks) lying within the CpG island were converted from CG to TG when treated with bisulphite, thereby implying that CIP2A promoter at these sites is unmethylated.

To initiate a systematic analysis of the mechanisms regulating CIP2A expression, 1.8 kb sequence upstream of the predicted CIP2A gene transcription start site was analyzed by using the Genomatix software. Figure 1A shows the predicted transcription factor binding sites, having matrix similarity of 95% and core similarity of 100%, on that genomic region. Genomatix software was also used to obtain the phylogenetic tree of the CIP2A promoter in different species (Fig. 1B). Interestingly, analysis of the −1500 to +450 bp region with MethPrimer software identified a CpG island between nucleotides −150 to +400 bp (blue shaded region in the Figure 1C). Importantly, alignment of the CIP2A promoters in different species also revealed conservation of CpG rich sequences on that region (Fig. S1A). Methylation of CpG sites in the regulatory regions of the genes is suggested to correlate with transcriptional silencing. Therefore, it was hypothesized that CIP2A expression in normal tissues and cell lines [20], [22], [23], [25] could be silenced due to promoter methylation. To this end, methylation status of −150 to +400 bp region was analyzed by using bisulphite sequencing. Genomic DNA samples collected for this analysis included freshly isolated cells from normal human blood, cultured human skin fibroblasts and cultured cancer cells (AGS and HeLa). Bisulphite treatment of the genomic DNA resulted in conversion of all cytosines in the sequenced promoter region to thymidines, in all samples (Fig. 1D and Fig. S1). Therefore, since methylation of CpG islands at CIP2A promoter was not observed in normal cells, it is unlikely that promoter de-methylation would explain increased CIP2A expression in cancerous cells.

### Functional and SNP analysis of CIP2A promoter

**Figure 2:**
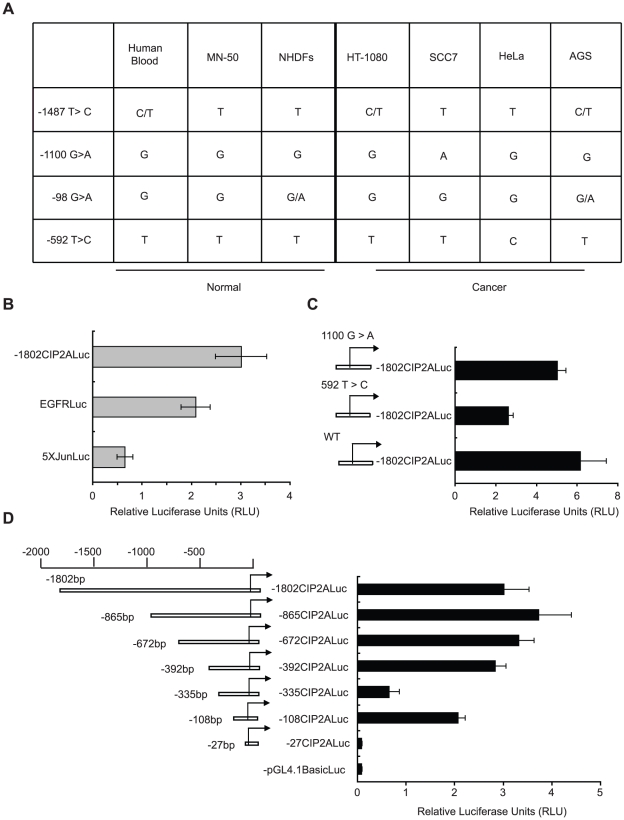
Functional and SNP analysis of CIP2A promoter. A. Identified single nucleotide polymorphisms on −1802 to +182 region of CIP2A promoter from indicated cells. Two cancer cell line specific changes were observed, namely G/A at −1101 in SCC-7 cell line and T/C at −592 in HeLa cell line. B. Relative Luciferase assay showing the relative activity of −1802 bp of CIP2A promoter in comparison to well known oncogenic promoters like EGFRLuc and 5xJunLuc. C. Luciferase assay showing comparison of activity between wild type (WT) CIP2A promoter and the indicated SNP mutants. D. Luciferase assay showing the activity of indicated CIP2A promoters. High basal activity is mediated by first 392 bp of the promoter, out of which, almost two-thirds of it was due to the first 108 bp. B–D, Shown is the Mean+SD from three independent experiments.

Single nucleotide polymorphisms (SNPs) have been reported to create novel transcription factor binding sites on the gene promoters. Specifically, a previous study showed that a SNP on MMP-1 promoter created a novel ETS binding site that augmented the MMP-1 transcription in cancer cells [27]. In order to assess the SNP status of CIP2A promoter, a region containing the CIP2A promoter depicted in Fig. 1A and exon 1 (−1802 bp to +182 bp) was sequenced from genomic DNA extracted from normal human peripheral blood, human non-malignant mononuclear monocytes (MN-50), normal human dermal fibroblasts (NHDFc), human fibrosarcoma cell line (HT1080), squamous cell carcinoma cell line (SCC7), cervical carcinoma cell line (HeLa) and gastric adenocarcinoma cell line (AGS). This analysis identified several SNPs on the analyzed region but only two of them (T>C at −592 in HeLa and G>A at −1100 in SCC7) were not found from any normal samples (Fig. 2A). However, as each of these two SNPs were found only from one cancer cell line, but not in the others analyzed, it is unlikely that they would create transcription factor binding sites that would augment CIP2A transcription generally in cancer. Of note, T>C at −592 in HeLa cells has not been previously documented in the databases.

In order to initiate functional characterization of CIP2A promoter, the −1802 bp to +182 bp 5′ upstream region of CIP2A gene that was analyzed for SNPs above, was cloned into pGL4.10 vector to create CIP2A promoter luciferase reporter construct (−1802CIP2ALuc). The promoter region was amplified from the genomic DNA of AGS cells, and this particular clone harboured nucleotides A at position −98 and T at the position −1487 (Fig. 2A). In order to estimate the relative transcriptional activity of the cloned CIP2A promoter fragment, we compared the luciferase activity of −1802CIP2ALuc to promoter/luciferase constructs known to be active in cancer cells. As shown in Fig. 2B, −1802CIP2ALuc activity was either equivalent or clearly higher than activity of EGFRLuc [28] or minimal 5xJunLuc [29] promoters respectively. Based on this result we concluded that the cloned −1802 bp to +182 bp 5′ upstream region of CIP2A gene contains an active CIP2A promoter.

Next the −1802CIP2ALuc construct was utilized to analyze whether the SNPs 592 T>C and 1100G>A identified above were functional. To this end, 592 T>C and 1100G>A mutations were introduced to −1802CIP2ALuc by site-specific mutagenesis and the activity of mutant constructs was compared to wild type 1802CIP2ALuc in AGS cells. On comparison to the wild type, there was no change seen in CIP2A luciferase activity with the 1100 G>A mutant clone, while 592 T>C mutant clone showed a marked decrease in the luciferase activity (Fig. 2C).

Finally, in order to characterize the regions at the CIP2A promoter that mediate its high transcriptional activity, several 5′-deletions of the promoter/reporter were cloned. Comparison of the basal activities of these deletion constructs in AGS cells revealed that regions between −392 and −1802 did not seem to contain transcription factor binding sites that would greatly contribute to high basal activity of CIP2A promoter (Fig. 2D). Interestingly, the high luciferase activity of −392CIP2ALuc was significantly reduced when additional 57 nucleotides were deleted resulting in −335CIP2ALuc construct (Fig. 2D). This seemed to be caused by exposure of a transcriptional repression domain, as further deletion of −335CIP2ALuc to −108CIP2ALuc resulted again in significantly increased luciferase activity (Fig. 2D). Intriguingly, this 108 bp CIP2A promoter accounted for more than 50% of the luciferase activity produced by the −1802CIP2ALuc (Fig. 2D).

Taken together, this data presents first functional analysis of CIP2A promoter region. Furthermore, these results strongly suggest that SNPs on CIP2A promoter do not significantly contribute to CIP2A overexpression in cancer.

### Regulation of CIP2A expression by the EGFR-MEK1/2 pathway

**Figure 3:**
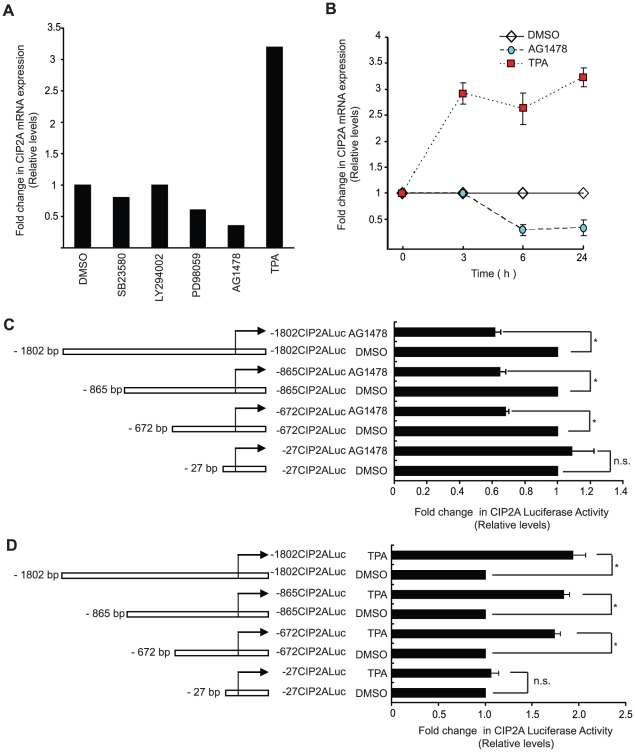
Regulation of CIP2A expression by the EGFR-MEK1/2 pathway. A. Quantitative PCR (qPCR) analysis showing levels of CIP2A mRNA extracted from AGS cells treated with DMSO, SB23580 (20 uM), LY294002 (10 uM), PD98059 (20 uM), AG1478 (10 uM) and TPA (100 nM) at 24 h time point. While a decrease in CIP2A mRNA level was observed with PD98059 and AG1478 treatments, TPA treatment augmented the CIP2A mRNA levels in AGS cells. B. qPCR analysis showing time-dependent regulation of CIP2A mRNA levels in AGS cells treated with DMSO, AG1478 (10 uM) and TPA (100 nM) for indicated time points. C and D Luciferase assays showing the activity of indicated CIP2A promoter deletions in cells treated either with DMSO, AG1478 (10 uM) or TPA (100 nM) for 24 h. (*, *P*<0.05; n.s. = non-significant). B–D. Shown is the Mean+SD from three independent experiments.

Results above suggested that CIP2A expression may be positively regulated by signaling pathways which stimulate its gene promoter activity. To address whether known oncogenic signaling pathways had role in regulating CIP2A expression, AGS cells were treated with well-defined chemical pathway inhibitors and activators. While inhibition of either p38 or PI3 kinases by SB23580 or LY294002, respectively, did not regulate in CIP2A mRNA levels, both MEK1/2 and EGFR inhibitors (PD98059 and AG1478) decreased CIP2A mRNA expression (Fig. 3A). Moreover, treatment of cells with phorbol ester TPA, a well-characterized activator of MEK1/2-ERK signaling pathway, increased CIP2A mRNA expression more than threefold (Fig. 3A). As shown in Fig. 3B, the effects of both AG1478 and TPA on CIP2A mRNA expression were already evident 6 hours post treatment which suggests a direct mode of action. In order to map the region on CIP2A promoter that is responsive to the EGFR-MEK1/2 pathway activity, cells transfected with various length of CIP2A luciferase promoter constructs were treated with AG1478 or TPA, and luciferase activity, as compared to control DMSO treatment, was measured as a read-out of promoter activity. As shown in Figs. 3C and D, AG1478 treatment decreased, while TPA treatment increased the luciferase activity from all except the shortest −27CIP2ALuc construct.

Taken together, these results show that CIP2A expression is positively regulated` by EGFR-MEK1/2 pathway and that the region responsive for the pathway activity lies between −27 bp and −672 bp on the CIP2A promoter.

### Characterization of MEK1/2 kinase responsive region on the CIP2A promoter

**Figure 4:**
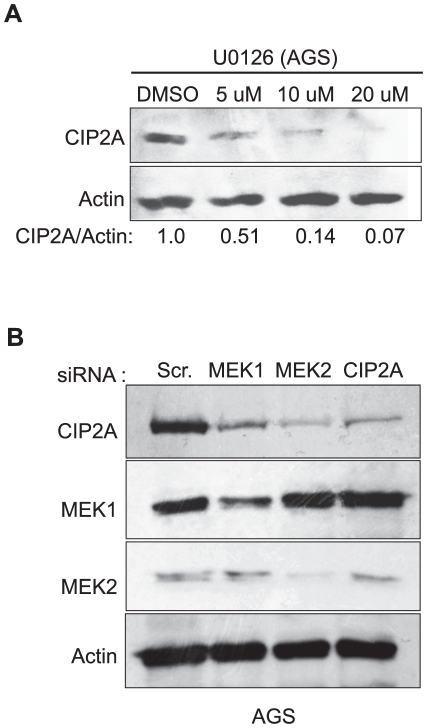
MEK1/2 kinases positively regulate CIP2A protein expression in human cancer cells. A. Western blot showing concentration dependent effect of specific MEK inhibitor, U0126, on CIP2A protein levels in AGS cells at 48 h time point. B. Western blot showing the effect of both MEK1 and MEK2 siRNAs on CIP2A protein levels in AGS cells at 72 h time point.

**Figure 5:**
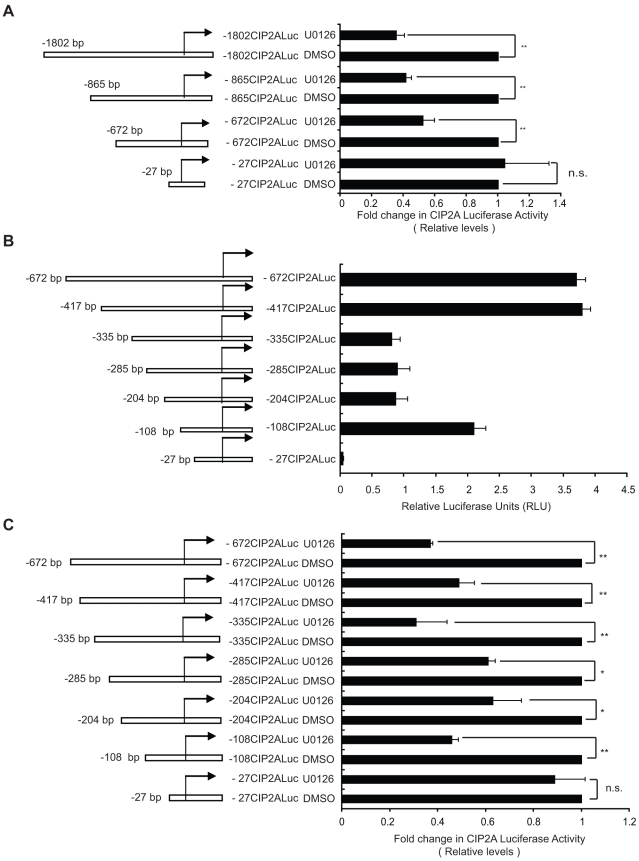
Characterization of MEK kinase responsive region on the CIP2A promoter. A. Luciferase assay showing the activity of different length CIP2A promoters when treated with DMSO and U0126 (10 uM) for 24 h, identifying the MEK responsive region to lie between −672 bp to −27 bp of the CIP2A promoter. B. Luciferase assay showing the basal activity indicated CIP2A promoters. C. Luciferase assays showing the activity of different length CIP2A promoters when treated with DMSO and U0126 (10 uM) for 24 h, further narrowing down the MEK responsive region to be between −108 bp and −27 bp of the CIP2A promoter (*, *P*<0.05; **, *P*<0.00; n.s. = non-significant). Shown is the Mean+SD from three independent experiments.

In order to validate the contribution of MEK1/2 kinases in the positive regulation of CIP2A expression, AGS cells were treated with UO126, a more specific and potent MEK1/2 inhibitor than the previously used PD98059, and CIP2A protein expression was studied 48 hours post treatment. As shown in Fig. 4A, UO126 dose-dependently decreased CIP2A protein expression. Furthermore, two different siRNAs against both MEK1 and MEK2 decreased CIP2A protein expression in AGS cells (Fig. 4B). Importantly the effects were not cell line specific as either chemical or siRNA-based MEK1/2 inhibition inhibited CIP2A protein expression also in PC-3 prostate cancer cell line (Fig. S2). To narrow down the MEK1/2-responsive region on the CIP2A promoter, AGS cells transfected with series of CIP2A luciferase reporter constructs were treated with UO126 (20 uM) and luciferase activities were measured 48 h post-treatment. In line with the results seen with AG1478 and TPA treatments, decreased luciferase activity of all of the other constructs except the −27CIP2ALuc was observed in UO126 treated cells (Fig. 5A). To further narrow down the MEK1/2-responsive region on the CIP2A promoter, various additional CIP2A luciferase promoter constructs were cloned (Fig. 5B). Comparison of the basal activities of these new constructs together with selected promoter constructs already analyzed in Fig. 2D, further confirmed that there are both repressive an activating promoter regions at CIP2A promoter between −27 bp and −400 bp (Fig. 5B). However, regardless of the basal activity of the reporter, UO126 treatment inhibited the activity of all reporters, including −108CIP2ALuc, with a notable exception of the −27CIP2ALuc construct (Fig. 5C). Therefore, these results demonstrate that MEK1/2 kinases positively regulate both CIP2A promoter activity and protein expression. Moreover, these results identify 81 bp between −108 bp and −27 as a MEK1/2-responsive region on CIP2A promoter.

### EGFR-MEK1/2 pathway regulates CIP2A expression through ETS1

**Figure 6:**
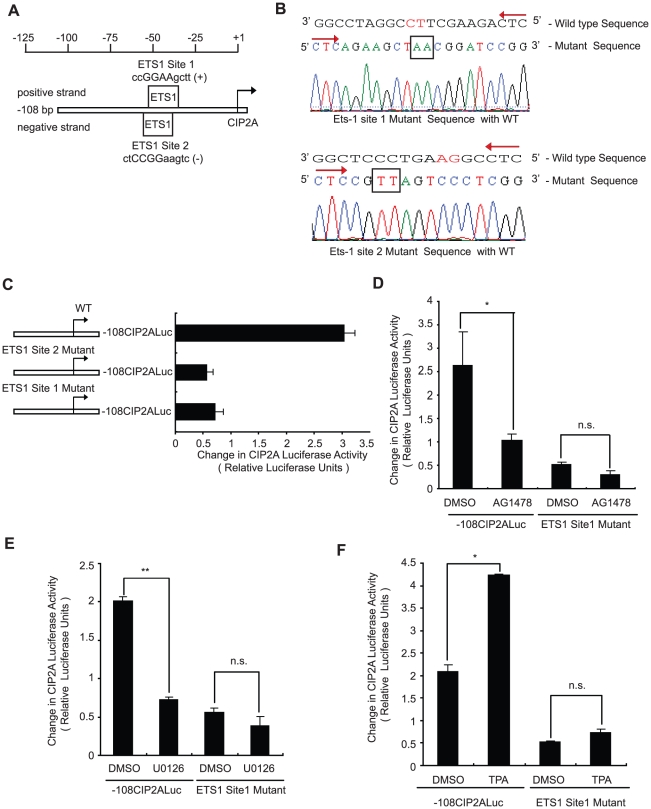
EGFR-MEK pathway regulates CIP2A expression through ETS-1. A. Schematic diagram of the 108 bp fragment of CIP2A promoter showing the location of two overlapping predicted ETS1 binding sites. B. Sequencing results of ETS-1 binding site mutants on the CIP2A promoter created using mutagenesis. The sequence on the top represents the wild type sequence (read from 5′ to 3′ end), while the sequence below is the mutated sequence (read from 5′ to 3′ end). The induced change in the sequence is shown within the rectangular box. C. Luciferase assay comparing the activity of either wild type −108CIP2ALuc or indicated ETS binding site mutant −108CIP2ALuc constructs. D,E&F. Luciferase assays comparing the activity of either wild type −108CIP2ALuc or ETS1 Site1 mutant −108CIP2ALuc constructs after treatment with DMSO, AG1478 (10 uM; D), UO126 (10 uM; E) or TPA (100 nM; F) for 24 h. (*, *P*<0.05; **, *P*<0.01; n.s. = non-significant). Shown is the Mean+SD from three independent experiments.

In order to identify the transcription factor(s) mediating the stimulatory effects of EGFR-MEK1/2 pathway in regulating CIP2A expression, we reverted back to the bioinformatic analysis done for the region between −27 bp to −108 bp (Fig. 1A). Out of the transcription factors predicted to bind to that region, ETS1 has an established role as a downstream effector of the MEK1/2 pathway [11], [12]. Therefore, we next created mutants for these ETS1 sites and compared the luciferase activity of the −108CIP2ALuc constructs harboring the mutations to that of the wild type. The two ETS sites and the mutation strategy are depicted in Fig. 6A, B. As shown in Fig. 6C, mutation of either of the ETS1 sites dramatically decreased the CIP2A promoter activity. To investigate whether ETS1 mediates the MEK1/2-dependent CIP2A regulation, cells transfected with both wild type and ETS1 mutant (site 1) −108CIP2ALuc constructs were treated either with AG1478, UO126 or TPA. While the wild type −108CIP2ALuc activity was significantly inhibited by AG1478 (Fig. 6D) or UO126 (Fig. 6E), and conversely activated by TPA (Fig. 6F), the ETS1 mutant promoter did not significantly respond to these treatments.

Taken together these results identify two functional ETS1 sites on the proximal CIP2A promoter. Moreover, these results strongly suggest that the MEK1/2-dependent stimulation of CIP2A promoter activity is mediated by ETS-family transcription factors.

### ETS1 binds to CIP2A promoter and regulates its expression in cancer cells

**Figure 7:**
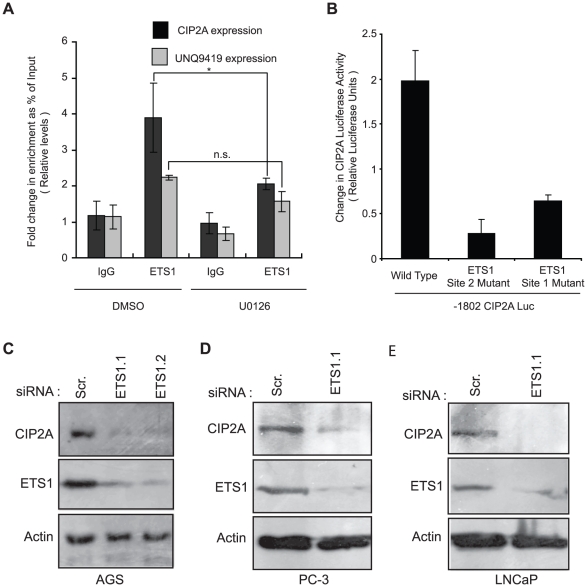
ETS-1 binds to CIP2A promoter and regulates its expression in cancer cells. A. Chromatin immunoprecipitation assay of ETS1 binding to CIP2A promoter in either DMSO or UO126 treated cells. UNQ9419 promoter is shown as a positive control. (*, *P*<0.05; **, *P*<0.01; n.s. = non-significant). B. Luciferase assay comparing the activity of either wild type −1802CIP2ALuc or indicated ETS binding site mutant −1802CIP2ALuc constructs. C,D&E. Western blot analysis of CIP2A and ETS1 protein levels in either AGS (C), PC-3 (D) or LNCaP (E) cells transfected with scrambled (Scr.) or ETS-1 specific siRNAs for 72 h.

To verify that ETS proteins do bind to the CIP2A promoter on MEK1/2 activity-dependent manner, we performed chromatin immunoprecipitation experiment with ETS1 antibody and amplification of the −66 bp to +20 bp fragment of the CIP2A promoter. As shown in Fig. 7A, ETS1 immunoprecipitation resulted in about 4-fold enrichment of CIP2A promoter occupancy as compared to the control IgG. Interestingly, in the same experiment CIP2A enrichment was even stronger than UNQ9419, an established ETS1 target that was used as a positive control [30]. Moreover, enrichment of CIP2A promoter was significantly inhibited by treatment of cells with U0126. Additionally, while the inhibition of CIP2A enrichment by UO126 was statistically significant, the inhibition of UNQ9419 enrichment was not (Fig. 7A).

Results above demonstrated that ETS elements mediate largely the basal and MEK-elicited activity of minimal −108CIP2ALuc promoter (Fig. 6). In order to establish whether these ETS elements are essential for full-length CIP2A promoter activity we mutated these sites on −1802CIP2ALuc and compared its activity to that of the wild type. As shown in Fig. 7B, mutation of either of the ETS1 sites dramatically decreased the −1802CIP2ALuc promoter activity. Additionally, in order to verify that ETS1 regulates endogenous CIP2A protein expression, two different siRNAs specific for ETS1 were transfected in AGS cells and cell lysates were immunoblotted 48 hours after for CIP2A, ETS1 and Actin levels. As shown in Fig. 7C, decreased CIP2A protein levels were observed with both of the ETS1 specific siRNAs. In addition, ETS1 positively regulated CIP2A expression in PC-3 (Fig. 7D) and LNCaP prostate cancer cell lines (Fig. 7E). Together, these results provide concrete experimental evidence of ETS1 being the transcription factor mediating EGFR-MEK1/2 dependent regulation of CIP2A in cancer cells.

**Figure 8:**
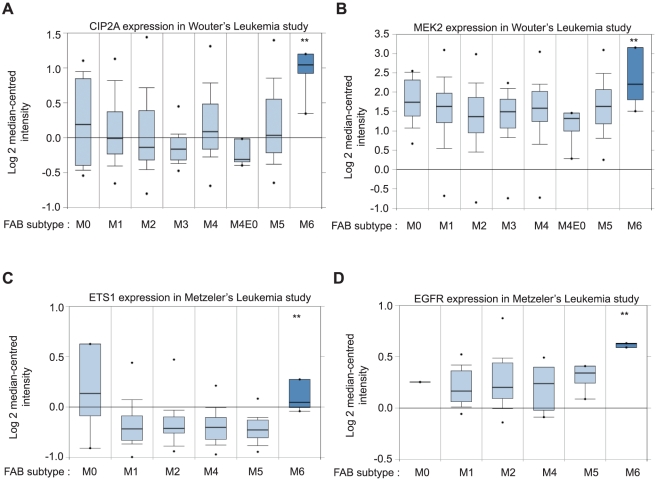
Bioinformatic analysis of CIP2A, MEK2, ETS1 and EGFR expression in acute myeloid leukemias. A,B,C&D. Oncomine database analysis of gene expression profiles for EGFR-MEK-ETS1 pathway genes revealed M6 subtype of acute myeloid leukemia as a cancer type in which CIP2A and representative genes of each level of the pathway are significantly upregulated (**,*P*<0.01).

Finally, in order to reveal whether the expression status of EGFR-MEK-ETS1 pathway components and CIP2A in human cancers, we referred to the Oncomine database (www.oncomine.org). This analysis revealed M6 subtype of acute myeloid leukemia as a cancer type in which CIP2A and representative genes of each level of the pathway (EGFR, MEK2 and ETS1) were significantly upregulated in two different genome wide leukemia studies [31], [32] (Fig. 8).

## Discussion

A recent systematic characterization of somatic mutations in 441 human tumors identified growth factor receptor signaling to MEK1/2-ERK pathway to be one of the most significantly altered pathways across human cancers [33]. Moreover, inhibition of oncogenic form of B-Raf in human malignant melanomas by a small-molecule inhibitor demonstrated very promising clinical efficacy already in phase I clinical trial [7]. Based on results of these studies and ample of earlier data demonstrating oncogenic function of EGFR-mediated MEK1/2-ERK pathway activation, it is evident that identification of oncogenic effectors of the pathway activity is important. In this study we demonstrate that CIP2A is a novel oncogenic target upregulated by EGFR-induced MEK1/2-ERK pathway activity. After its original cloning [34], and further functional characterization [20], CIP2A has been demonstrated to promote malignant cell growth by using various human cancer cell models [20], [22], [23], [24], [25]. Moreover, the CIP2A protein has been shown to be overexpressed with very high frequency in different types of human malignancies. Except human breast cancer where CIP2A is overexpressed in 40% of patient samples [22], in all other studied cancer types the frequency is between 65 to 87% of patients [20], [23], [24], [25]. This makes CIP2A overexpression together with MEK1/2-ERK pathway activation as one of the most frequent alterations in human cancers. However, prior to this study, the mechanisms by which CIP2A expression is induced in human cancer cells have been very poorly understood.

In order to identify mechanisms responsible for high basal expression of CIP2A in human cancer cells, we have in this study systematically analyzed contribution of several potential gene regulatory mechanisms that have been earlier shown to affect gene expression in human cancers. Although we have not exclusively ruled out that either promoter methylation or functional SNPs at the CIP2A promoter might contribute to high CIP2A expression in cancer, our data does indicate that these mechanisms most probably are not relevant for regulation of the CIP2A promoter activity. In order to identify promoter regions functionally implicated in regulation of CIP2A expression in cancer cells, we created altogether 10 promoter deletion reporter constructs. Data shown in figures 2D and 5B allowed us to conclude that the promoter region between −335 bp and −392 bp contains a strong activating promoter element, whereas region between −108 bp and −204 bp contains a strong repressor element. Furthermore, the data shows, that promoter region upstream of −417 bp do not significantly contribute to the high basal activity of the studied CIP2A promoter in AGS cells, whereas, promoter region between −27 bp and −108 bp has a very strong activating element accounting for at least 50% of the total activity of the −1802 bp promoter. In addition to promoter regulation, another important mechanism that contributes towards protein expression is modification of its turn over rate or stability. CIP2A has been previously shown to be very long lived protein in hepatocellular carcinoma cell line [26]. Therefore, elucidation of mechanisms contributing towards CIP2A stability in cancer cells will be a relevant question to be addressed in the future.

Our subsequent experiments with the CIP2A promoter identified two partly overlapping ETS-binding sites between −60 bp to −30 bp to be largely responsible for high activity of the full-length promoter. Interestingly, it's been shown that over two-thirds of the 27 genes of the human ETS family can be co-expressed in a given cell type [35]. Moreover, in principle all ETS proteins can recognize the same 5′-GGA(A/T)-3′ motif within a particular promoter [35]. This extensive co-expression and conservation of the ETS DNA binding sites, makes matching a particular ETS protein to a specific promoter a challenge. However, results of our siRNA experiments in three different cancer cell lines show positive regulation of CIP2A expression by ETS1 (Fig. 7C,D,E). Moreover, our chromatin immunoprecipitation experiments, using an ETS1 specific antibody, revealed a 4 fold enrichment of CIP2A promoter occupancy (Fig. 7A). In addition, the results of a recent study [36], demonstrating binding of ETS1 on CIP2A promoter in Jurkat T-cell leukemia strongly supports our main conclusion that ETS1 specifically is involved in positive regulation of CIP2A. However, due to the cell type specificity and co-operative DNA binding between the ETS family members, possibility of other ETS family members also regulating CIP2A expression cannot be ruled out. Therefore, it is evident that further analysis of the functional relevance of the other predicted transcription factor binding sites, and other ETS family members, in CIP2A regulation (Fig. 1A), will be an important future research goal.

Many earlier studies have shown that ETS1 promotes, in addition to invasion related processes [16], also cellular transformation [17], [18], [19]. Regardless of this, the ETS1 target genes supporting cellular transformation are poorly identified. In this study, we validate CIP2A as a novel ETS1 target gene. As described above, CIP2A has been shown to promote proliferation and anchorage independent growth of various cancer cell lines and siRNA-mediated CIP2A depletion has been shown to very potently inhibit xenograft tumor growth [20], [22]. Moreover, overexpression of CIP2A promotes human cell transformation [20]. However, both others and we have shown that CIP2A knockdown does not affect cancer cell migration or invasion through matrigel [20], [23]. Taken together, these results may allow us to speculate that positive regulation of CIP2A contributes to the less explored ETS1 driven processes such as capacity to grow in an anchorage independent manner and cellular transformation. Both ETS1 and CIP2A have been associated with clinical aggressivity in breast cancer [22], [37]. Our own bioinformatics analysis of overexpression of CIP2A and components of EGFR-MEK1/2-ETS1 pathway revealed M6 subtype of acute myeloid leukemia (AML) as a cancer type in which CIP2A and representative genes of each level of the pathway are significantly up regulated (Fig. 8) [31], [32]. Importantly, CIP2A overexpression in AML patients compared to healthy controls was recently verified both at mRNA and protein level [38].

In summary, this work provides first systematic analysis of mechanisms regulating expression of a newly characterized human oncoprotein CIP2A. Our results demonstrate that EGFR-MEK1/2-ETS1 pathway is a critical positive regulator of CIP2A expression. Thereby these results reveal a potential link between deregulated EGFR-MEK1/2-ETS1 pathway signaling and CIP2A-dependent tumor growth. Importantly, in addition to its scientific impact, this work also provides several important resources for future studies aiming at characterizing mechanisms that regulate CIP2A expression both in pathological as well as physiological situations.

## Materials and Methods

### Plasmid constructs

The upstream region of the CIP2A promoter containing exon 1 (−1802 bp to +182 bp) was amplified by PCR from the genomic DNA of AGS cells, and the fragment was cloned into pGL4.10-Basic vector (Promega, Madison, WI, U.S.A) between XhoI and Bgl II restriction enzyme sites. Then various length luciferase promoter constructs were created using the Deletion Kit for Kilo-Sequencing (Takara Bio Inc., Japan) as per the manufacturer's instructions. All constructs were sequenced before use.

### Transient transfections of plasmids and luciferase assay

All cell lines were obtained from ATCC. AGS cells were plated in each well of the 96-well plate on day one. Next day respective luciferase reporter construct was transfected using Fugene (Roche Diagnostics, IN, U.S.A) according to the manufacturer's directions. To normalize the luciferase activity, a control plasmid expressing Renilla luciferase sequence was co-transfected into the cells. Cells were then analysed 48 h post-transfection using the Dual – Glo Luciferase Assay System (Promega, Madison, WI, U.S.A). Results are presented post normalization with the Renilla luciferase levels. EGFRLuc (region −1109 to −16) was a kind gift by Dr. Micheal Birrer, and 5xJunLuc containing jun2 element from the c-*jun* promoter in front of the thymidine kinase promoter (TK) was a kind gift by Dr. Hans Van Dam [29].

### DNA Extraction and Bisulphite sequencing

Genomic DNA of the cell lines and blood sample was extracted using DNA isolation kit (Nucleon™ BACC Genomic DNA Extraction Kit, GE Healthcare Europe GmbH). Bisulfite modification of genomic DNA was carried out by using EZ DNA methylation kit (Zymo Research, Orange, CA) according to the manufacturer's instructions. Primers are described in Table S1.

### qRT-PCR

mRNA was extracted from cells by using RNeasy kit (Qiagen, Valencia, CA) and converted to cDNA by using M-MLV Reverse Transcriptase kit (Promega, Madison, WI, U.S.A.). cDNAs were subjected to quantitative real-time PCR by using Light Cycler (Roche Diagnostics, IN, U.S.A.) and SYBR Green PCR Master Mix kit (Roche Diagnostics, IN, U.S.A) as described previously [24]. Primers are described in Table S1.

### Mutagenesis

Mutagenesis for CIP2A promoter constructs was done using QuickChange site directed mutagenesis kit from Stratagene (La Jolla, CA, USA). Primers are described in Table S1. Mutations were verified by sequencing and two individual clones for each mutation was used to verify results.

### siRNA experiments

HP Validated siRNAs for human MEK1/2 and ETS1 were purchased from (Qiagen Technologies). Cells were transfected with 100 pmoles of siRNA per well in a six-well plate using Lipofectamine 2000 Reagent (Invitrogen, Carlsbad, CA) in antibiotic free growth medium, as per the manufacturer's instructions. Cells were harvested, and mRNA was extracted 24 h posttransfection. For Western Blots, cells were harvested and lysates prepared 72 h posttransfection. siRNA sequences are described in Table S1.

### Chromatin immunoprecipitation

The ChIP procedure was performed as described previously [39] with AGS cells grown to 70–80% confluence. The chromatin was sheared to an average size of 200–500 bp. After cross-linking reversal and proteinase K digestion, each individual IP was purified with the use of a QIAquick PCR purification kit (Qiagen, Valencia, CA,USA), and samples were eluted with 50 µl of elution buffer. After elution the IPs were examined by gene-specific qPCR. Primers are described in Table S1. The antibodies used were ETS1 (C20) and control IgG both from Santa Cruz, Biotechnology, Santa Cruz, CA, USA).

### Protein extraction and western blotting

Proteins were extracted in hot Laemmli sample buffer and subjected to Western blot analysis. 30 µg total protein extracts were separated by 12% SDS-PAGE and transferred to nitrocellulose membranes. Membranes were blocked with 5% non-fat milk in TBS-0.1%-NP40 and then incubated with mouse monoclonal MEK1 (H8), the mouse monoclonal MEK2 (A-1), the rabbit polyclonal ETS1 (C20), goat polyclonal anti-β-Actin (all from Santa Cruz, Biotechnology, Santa Cruz, CA, USA) or with rabbit polyclonal anti-CIP2A [34].

### Statistical Analysis

Statistical significance was calculated using two-sided Student t-test (SPSS Inc.) and included in the respective figure legend.

## Supporting Information

Figure S1 **Methylation Status of CIP2A Promoter.** A. Diagram shows alignment of CIP2A promoter in various lower species showing conserved CpG sites (represented by rectangular boxes) in them. B,C,D&E. Shows the sequencing results of the extracted genomic DNA from normal human blood (B), normal human dermal fibroblasts (C), AGS cells (D) and HeLa (E) cell lines. All CpG sites (represented by black rectangular blocks) lying within the CpG island were converted from CG to TG when treated with bisulphite, thereby implying that CIP2A promoter is not methylated at these sites.
(EPS)

Figure S2 **MEK1/2 kinases positively regulate CIP2A protein expression in human cancer cells.** A. Western blot showing effect of specific MEK inhibitor, U0126, on CIP2A protein levels in PC-3 cells at 48 h. B&C. Western blot showing the effect of both MEK1 (A) and MEK2 (B) siRNAs on CIP2A protein levels in PC-3 cells at 72 h time point. CIP2A expression was reduced by both MEK siRNAs.
(EPS)

Table S1 **Sequences of primers and siRNAs.**
(EPS)
